# Cost-effective reconstruction of long-standing lateral orbital rim defect with bone-prelaminated forehead flap using iliac crest: A case report

**DOI:** 10.1097/MD.0000000000050181

**Published:** 2026-08-14

**Authors:** Ahmed Al-Marrawi, Hussein Hammadeh, Mohammad Alaa Aldakak, Mhd Husam Alhilbawi

**Affiliations:** a Department of Plastic Surgery, Al-Mouwasat University Hospital, Faculty of Medicine, Damascus University, Damascus, Syrian Arab Republic; b Faculty of Medicine, Damascus University, Damascus, Syrian Arab Republic.

**Keywords:** case report, crane flap, iliac crest bone graft, lateral orbital rim defect, prelaminated forehead flap

## Abstract

**Rationale:**

Reconstructing composite lateral orbital rim defects after high-energy trauma remains challenging, particularly after infected alloplastic failure and in settings where microsurgery or patient-specific implants are unavailable. Autologous regional reconstruction may restore soft tissue coverage and bony support while avoiding additional hardware.

**Patient concerns:**

A 33-year-old man presented with a long-standing post-traumatic right orbital deformity after ballistic injury, including globe loss and composite defects of the lateral orbital rim, temporal bone, and adjacent soft tissues. A previously placed metallic mesh had become exposed and infected, causing pain, deformity, and an inability to proceed with prosthetic rehabilitation.

**Diagnoses:**

Infected exposed alloplastic reconstruction with an anophthalmic right orbital deformity and composite lateral orbital rim, temporal, and soft tissue loss.

**Interventions:**

Management was staged. After mesh removal, culture-directed infection control, crane flap preparation, delayed transfer, supragaleal retrieval, and split-thickness skin grafting, definitive repair was performed using a contralateral forehead flap measuring 6 cm × 6 cm with a 12-cm pedicle, prelaminated with an iliac crest bone graft in a narrow distal subcutaneous pocket. The flap reconstructed the lateral orbital rim, and lateral canthal support was restored using nylon loop-suture suspension anchored through the graft, without rigid alloplastic hardware.

**Outcomes:**

Distal venous congestion resolved after superficial suture release and leech therapy, without necrosis. Early imaging confirmed stable graft positioning, and the socket was anatomically suitable for future ocular prosthesis placement; however, prosthetic fitting had not yet been completed.

**Lessons:**

This technique may represent a cost-conscious option in resource-limited settings, but longer follow-up is needed.

## 1. Introduction

Orbital fractures are common in craniomaxillofacial trauma, accounting for approximately 10% to 25% of facial injuries.^[1,2]^ Their high frequency reflects the exposed position of the midface and the thin bony walls of the orbit, and they can lead to substantial functional impairment.^[3,4]^ The most common etiology is violent assault or nonviolent traumatic injury (49.4%), and the most frequently involved site is the zygoma (23.6%), followed by the orbital floor (21.4%), maxilla, mandible, and nasal bones.^[1,5]^

Orbital reconstruction after trauma is technically demanding because of restricted surgical exposure and complex anatomy, with critical neurovascular structures nearby.^[6]^ Repair aims to reduce herniated orbital contents, remove unstable bone, and restore the bony framework while preserving extraocular motility and facilitating functional and aesthetic rehabilitation.^[7]^ Available reconstructive materials include titanium mesh and autologous bone grafts, each with distinct advantages and drawbacks; no single material is universally superior, and selection should be individualized according to defect pattern, tissue quality, prior infection, resource availability, and patient-specific goals.^[8]^

To support the novelty of this technique, we performed a targeted PubMed search using combinations of the terms “prelaminated flap,” “pre-laminated flap,” “prelamination,” “pre-lamination,” “bone prelamination,” “bone pre-lamination,” “forehead flap,” “orbital rim,” “lateral orbital rim,” “orbital reconstruction,” and “craniofacial reconstruction.” This search yielded no previously reported case describing the use of a bone-prelaminated pedicled forehead flap to bridge a lateral orbital rim defect without rigid alloplastic hardware.

Here, we present a staged, cost-conscious autologous reconstruction of a long-standing post-traumatic lateral orbital rim defect after ballistic injury in a resource-limited setting. The reconstruction used crane flap preparation followed by a prelaminated contralateral forehead flap (6 cm × 6 cm; 12-cm pedicle) containing an iliac crest bone graft, combined with subcutaneous loop-suture lateral canthus suspension. To our knowledge, this is the first reported case in which a bone-prelaminated contralateral forehead flap was used to reconstruct and bridge the lateral orbital rim without additional rigid hardware, while also providing an autologous anchor for lateral canthal support.

## 2. Case presentation

**Figure 1.**
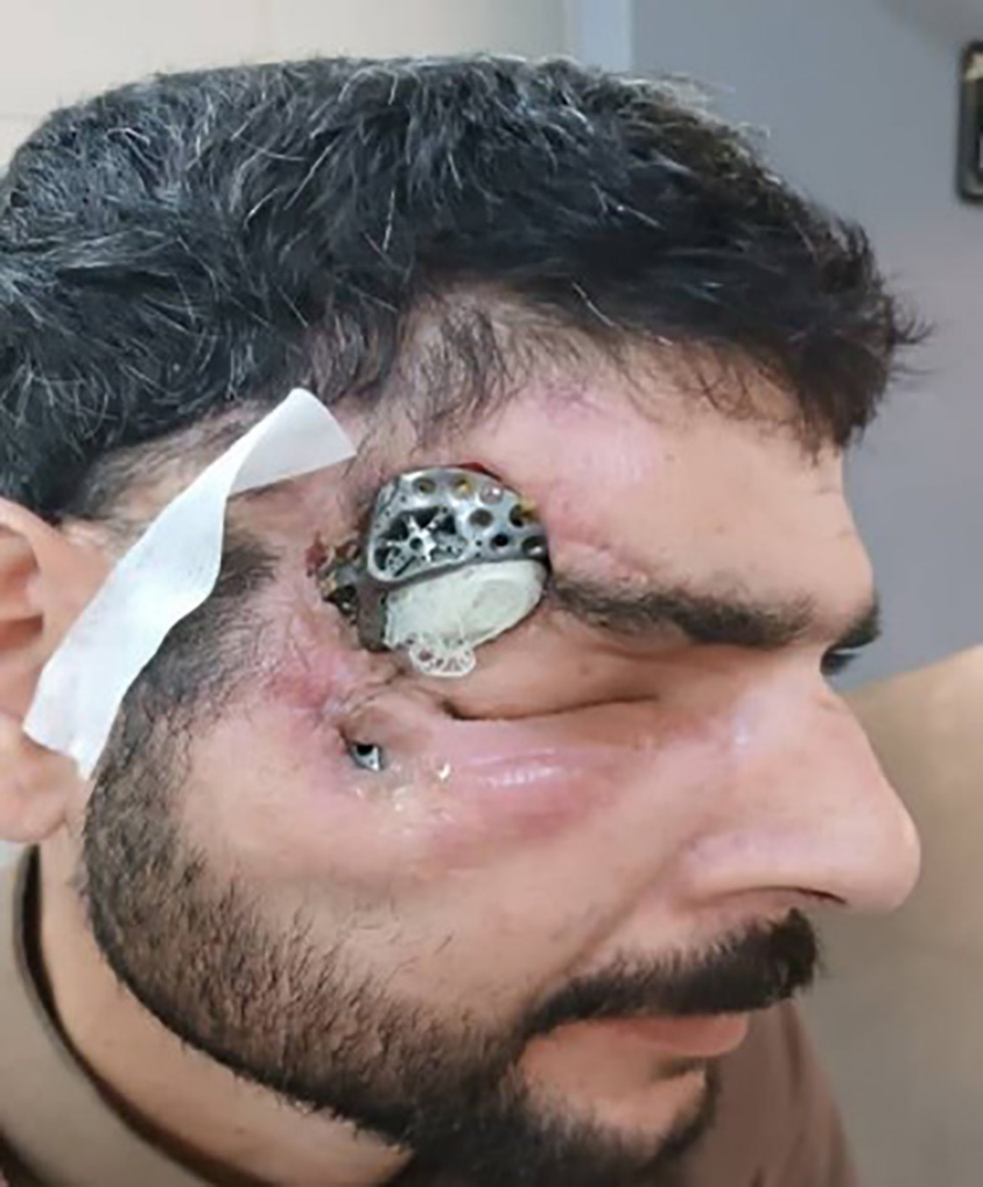
Clinical photograph demonstrating exposure of the previously implanted metallic mesh at the right lateral orbital region with surrounding soft tissue swelling and localized infection.

A 33-year-old Syrian male presented to the plastic surgery clinic with a post-traumatic deformity of the right orbit following a gunshot injury sustained 14 years earlier during the Syrian conflict. The initial trauma resulted in complete loss of the right globe and a large composite defect involving the lateral orbital rim, temporal bone, and adjacent soft tissues. The patient had undergone several unsuccessful reconstructive attempts at other centers, including placement of a metallic mesh, which later became exposed and infected (Fig. 1). At presentation, he reported localized pain, deformity, and extrusion of the infected mesh. His main goal was to obtain sufficient orbital support and soft tissue stability to allow future ocular prosthesis fitting. His past medical history was unremarkable, with no systemic comorbidities or previous chronic illnesses reported.

### 2.1. Crane principle

**Figure 2.**
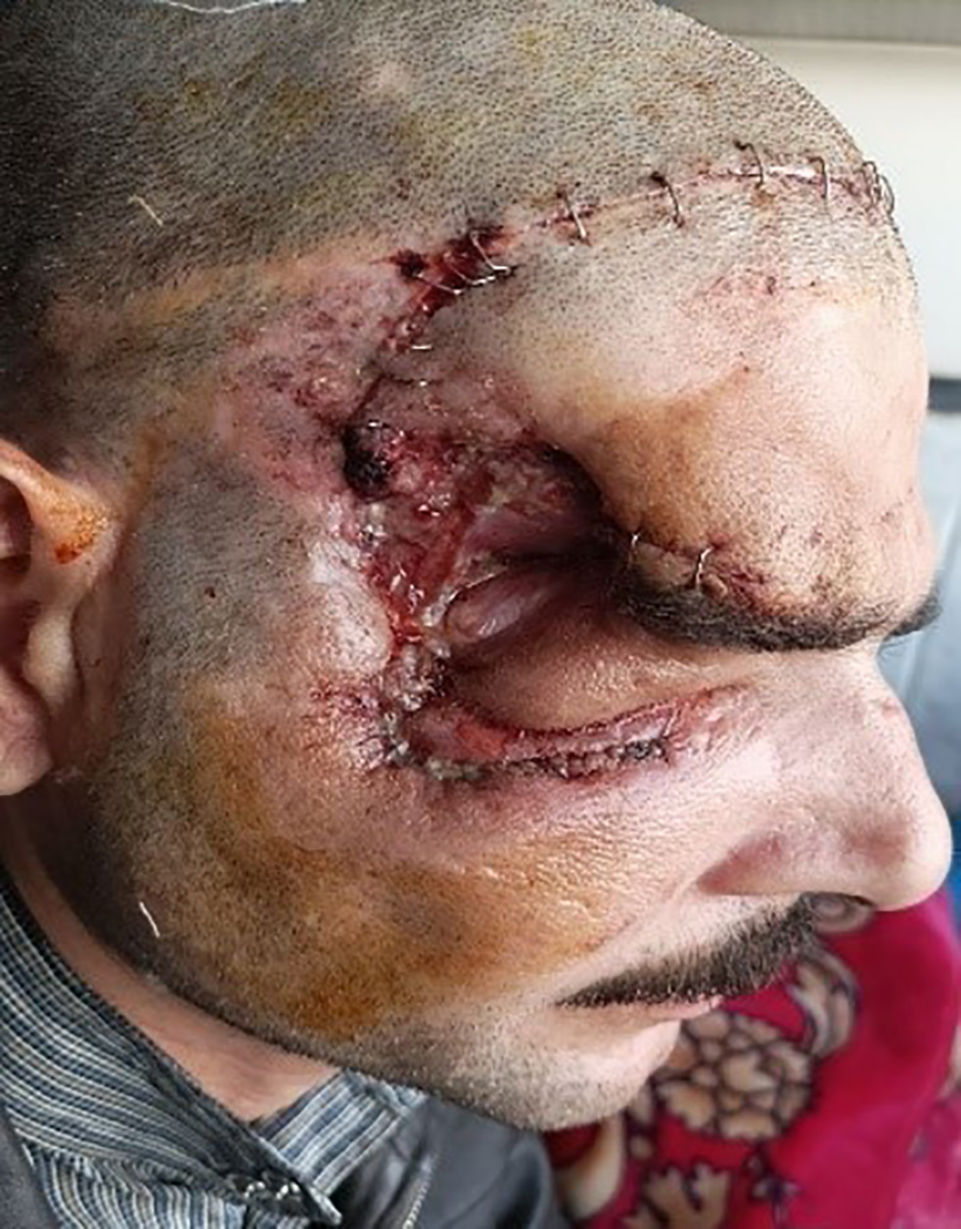
View following the removal of the infected metallic mesh, showing exposed bone at the lateral orbital region. A delayed crane flap was designed in the same setting for subsequent coverage.

**Figure 3.**
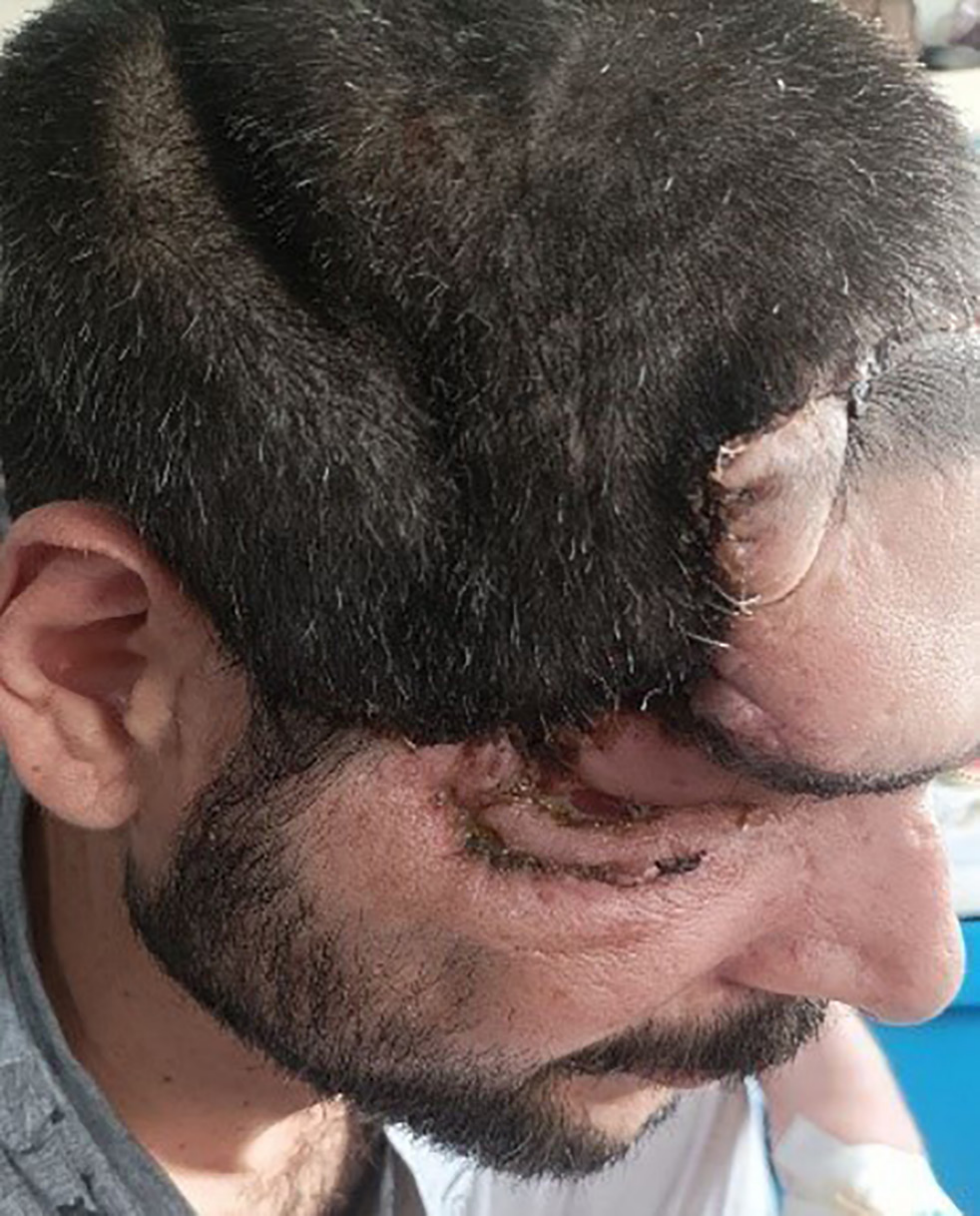
Transfer of a crane flap to cover the exposed temporal bone and secure soft tissue protection over the defect.

**Figure 4.**
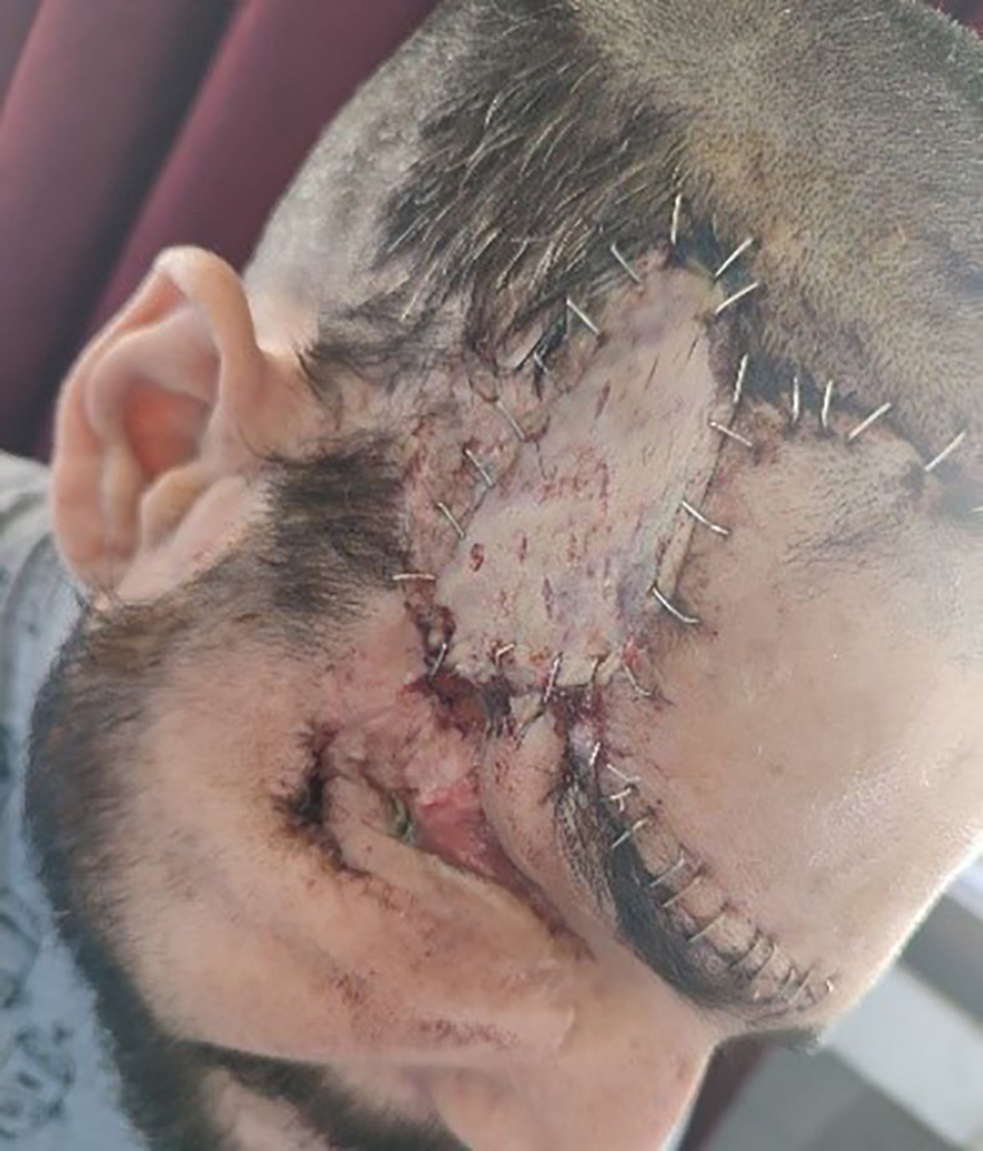
Granulation galea coverage of the temporal defect with a split-thickness skin graft harvested from the thigh.

The first stage involved the removal of the infected metallic mesh, followed by wound culture and culture-directed infection control. Once local infection had improved, coverage of the exposed residual temporal bone at the lateral orbital region was planned using the crane flap principle. A delayed crane flap was therefore designed in the same setting and elevated 2 week later for transfer (Fig. 2). The flap was then transferred to cover the exposed temporal bone, providing soft tissue protection over the defect (Fig. 3). The donor site was not grafted immediately; instead, a tie-over bandage was applied to preserve the hair-bearing scalp and allow granulation tissue formation. Three weeks later, retrieval of the flap was performed by supragaleal dissection, and the flap was returned to its donor site as skin and connective tissue, thereby preserving hair follicles and scalp integrity. The vascularized galea left behind successfully covered the temporal defect, which was then resurfaced with a split-thickness skin graft harvested from the thigh (Fig. 4). Both the flap and the skin graft survived completely without complications.

### 2.2. Definitive reconstruction: prelaminated forehead flap with iliac bone graft

After achieving stable soft tissue coverage and infection control, definitive reconstruction of the lateral orbital rim was planned. Because the ipsilateral forehead and periorbital region had been affected by war-related trauma, scarring, and prior infection, and because Doppler assessment did not clearly identify a reliable ipsilateral arterial pedicle, a contralateral forehead flap was selected. This was considered safer, particularly given the patient’s heavy smoking history and the need for a long, reliable vascularized flap.

**Figure 5.**
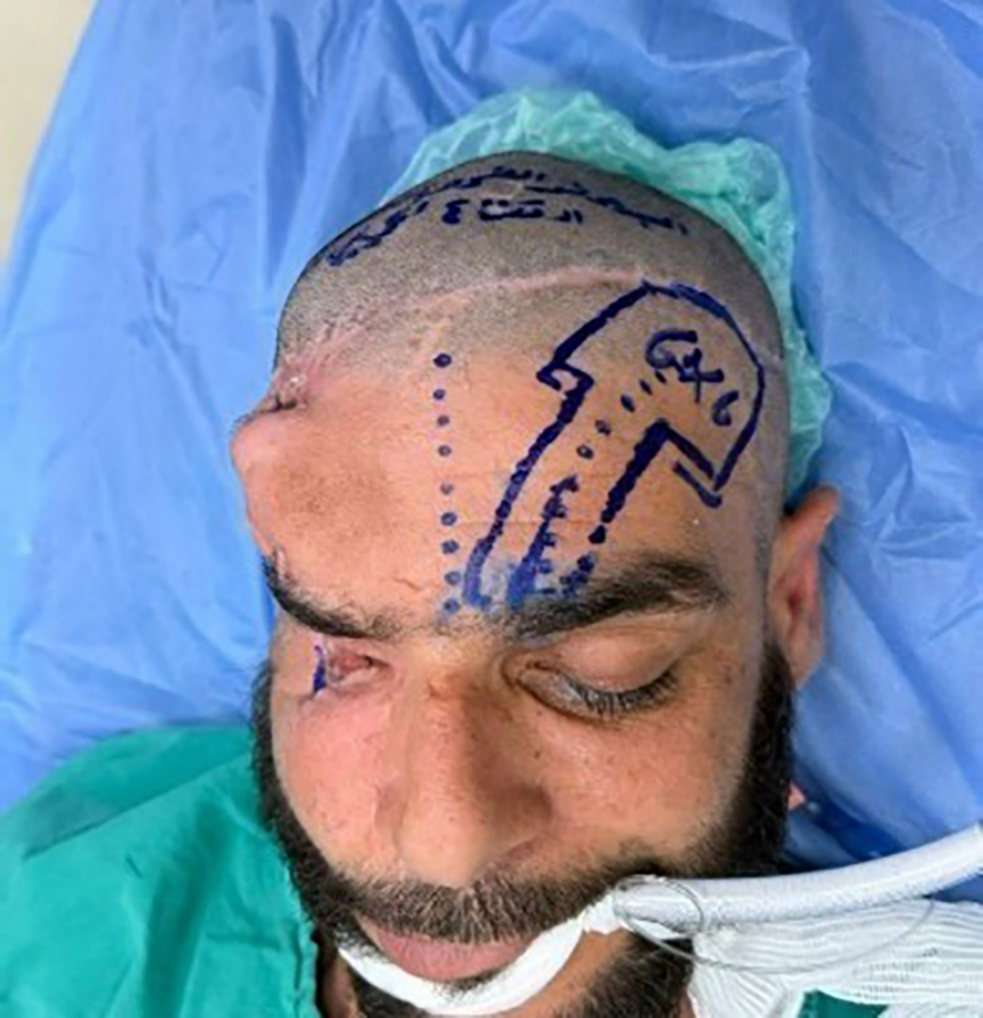
Design of a large contralateral forehead flap (6 cm × 6 cm) after identifying the supratrochlear artery to serve as the pedicled flap for lateral orbital rim reconstruction.

A large contralateral forehead flap measuring 6 cm × 6 cm, with a pedicle length of approximately 12 cm, was designed based on the contralateral supratrochlear artery (Fig. 5). An iliac crest bone graft was harvested and contoured into a thinned half-cylinder shape to improve adaptability to the orbital rim defect. The graft was harvested without periosteum. During flap prelamination, the bone graft was inserted into a very narrow subcutaneous pocket beneath the distal portion of the flap. The graft was placed in direct contact with the underlying galeal/frontalis vascular bed to promote early neovascular ingrowth during the prelamination interval. The pocket was intentionally created only large enough to accommodate the graft and prevent graft mobility; therefore, the graft was not fixed with sutures, and its stability depended on the tight fit within the pocket.

**Figure 6.**
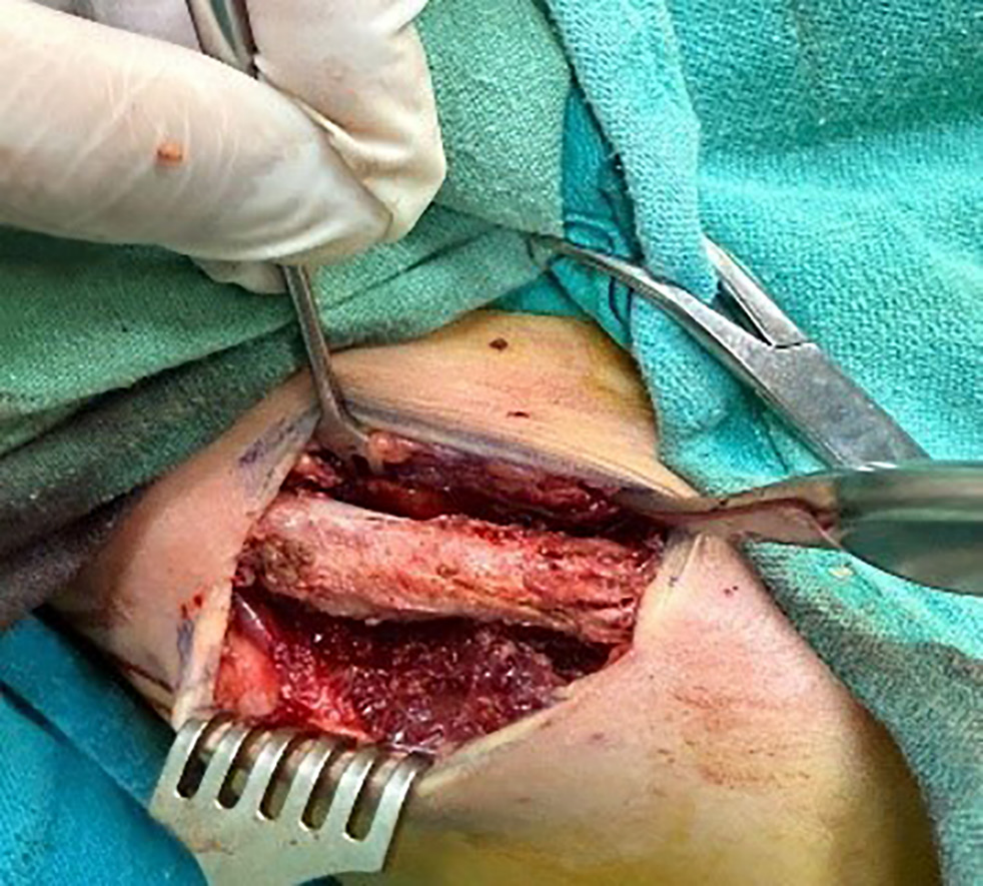
Harvesting an iliac crest bone graft to be used for lamination beneath the distal portion of the forehead flap.

The graft was placed horizontally relative to the forehead flap surface so that, after flap transfer and approximately 270° rotational twist of the pedicle, it would assume a near-vertical orientation appropriate for lateral orbital rim reconstruction. This represented a true pedicle twist rather than a simple 180° transposition arc. Therefore, potential pedicle kinking and venous outflow compromise were recognized as technical risks of this configuration. The proximal and middle portions of the flap were dissected, while the distal portion was left intact to preserve vascularity and create the prelamination pocket (Fig. 6).

**Figure 7.**
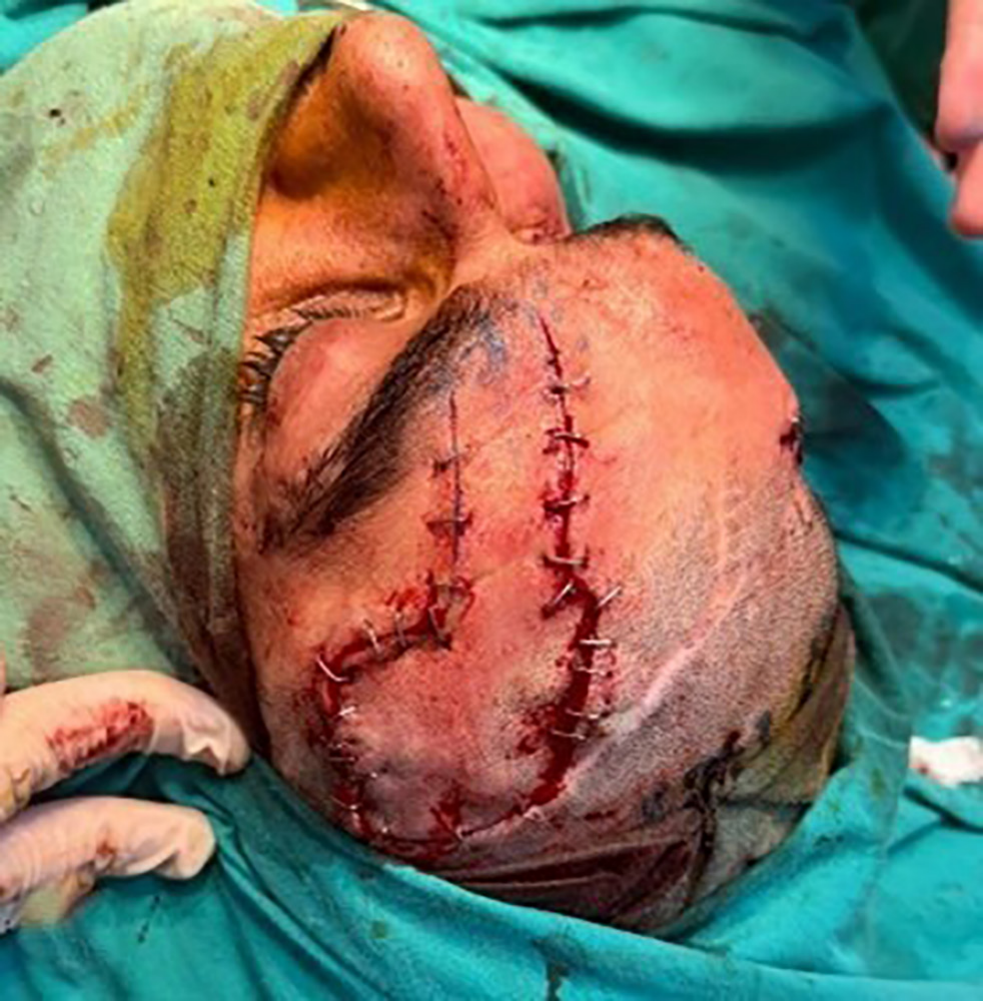
Postoperative view demonstrating a delayed prelaminated forehead flap following the incorporation of the iliac crest bone graft.

**Figure 8.**
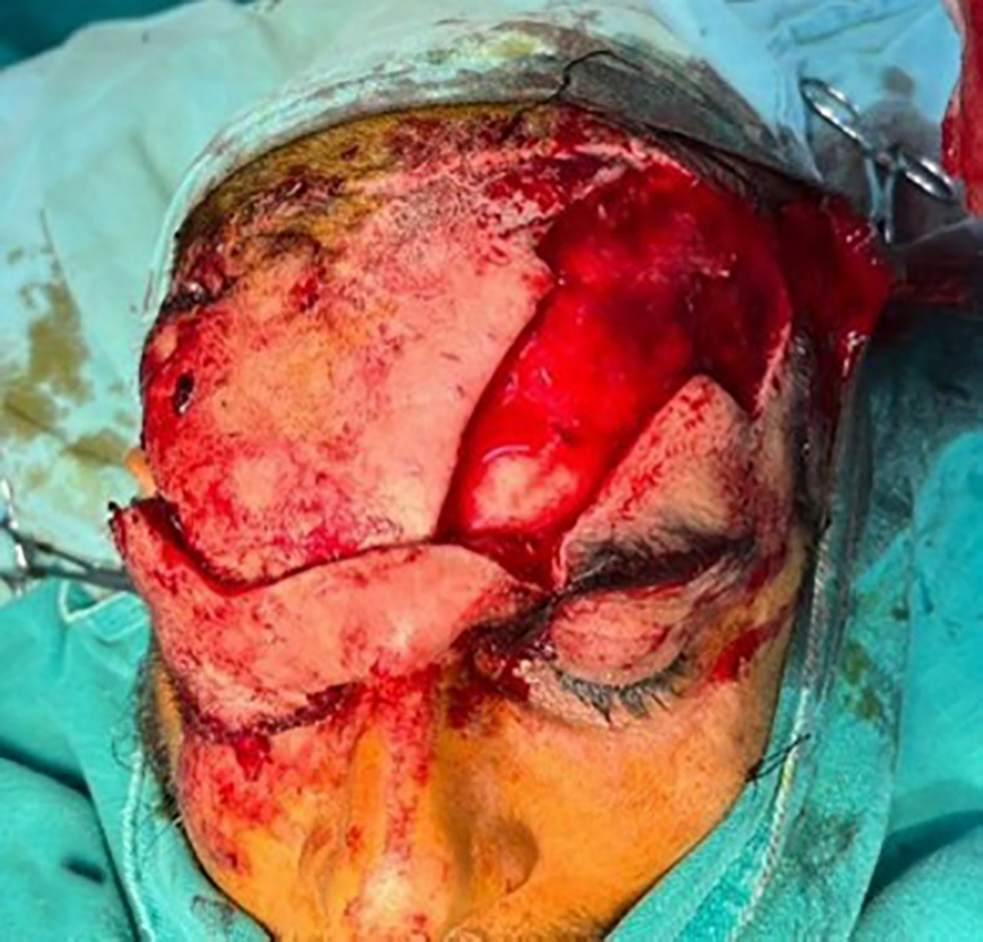
Elevation of the laminated forehead flap and transposition to the defect site for the reconstruction of the right lateral orbital rim.

**Figure 9.**
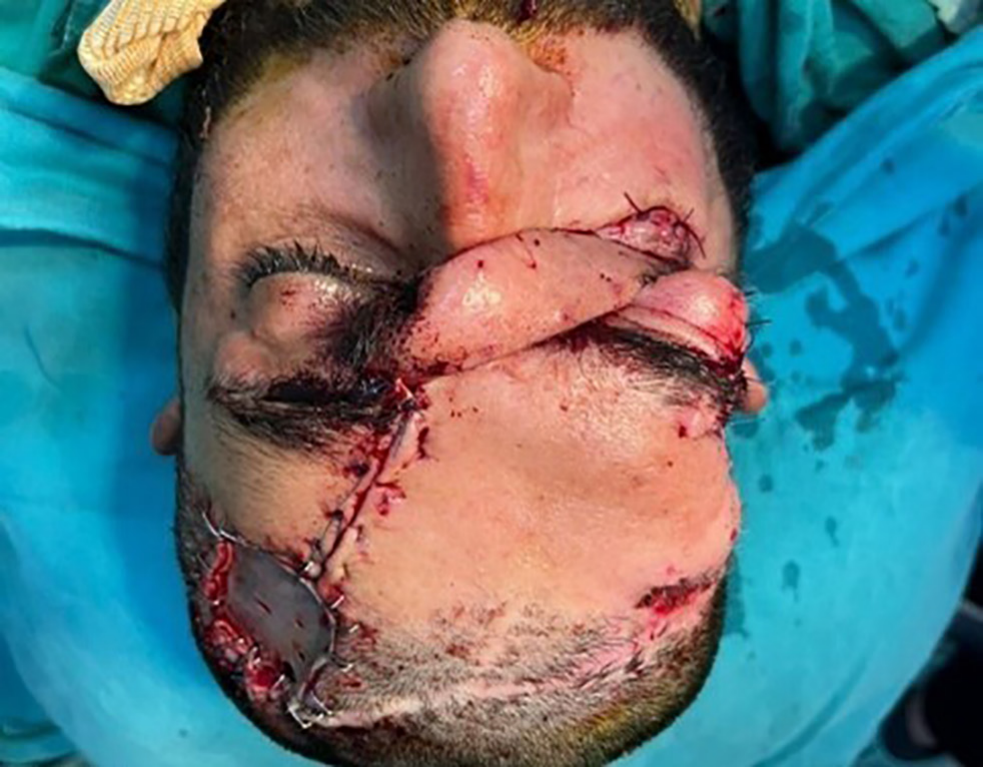
Transposition of the laminated forehead flap to reconstruct the right lateral orbital rim, with the remaining uncovered defect resurfaced using a split-thickness skin graft (STSG). STSG = split-thickness skin graft.

The postoperative appearance of the delayed prelaminated flap following bone incorporation is shown in Figure 7. After 1 week of delay, the laminated forehead flap was elevated and transposed to the lateral orbital rim defect (Fig. 8). The forehead donor site was closed primarily; a small segment that could not be closed directly was resurfaced with a split-thickness skin graft placed over the closure line (Fig. 9).

**Figure 10.**
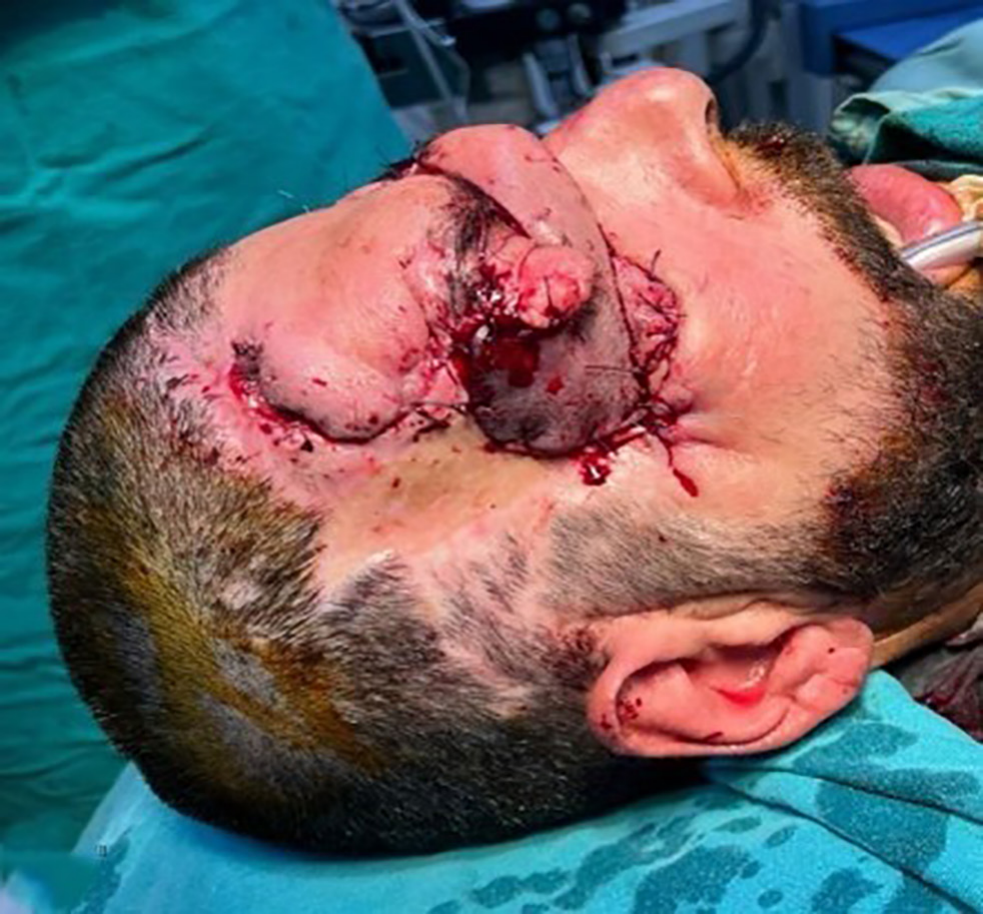
Venous congestion observed at the distal portion of the transferred flap, which was successfully managed with leech therapy.

During the first 24 hours after flap transfer, venous congestion developed at the distal portion of the flap. This was identified clinically by distal color change, swelling/tension, and increased congestion on compression, while capillary refill remained preserved. The congestion was thought to be multifactorial, related to partial flap elevation during prelamination, the tight subcutaneous pocket around the bone graft, the true approximately 270° pedicle twist, and the resulting mild pedicle compression. The patient’s heavy smoking history may also have contributed to impaired venous outflow. Several superficial sutures were loosened within the first 24 hours to reduce tension and improve venous return. Medicinal leech therapy was then applied to the congested distal portion of the flap for approximately 4 days, using 2 to 3 leeches per session; each leech was allowed to detach spontaneously after feeding. Prophylactic levofloxacin was administered during leech therapy. The congestion resolved without partial flap necrosis and did not delay subsequent reconstructive stages (Fig. 10).

### 2.3. Flap separation and lateral canthus suspension

**Figure 11.**
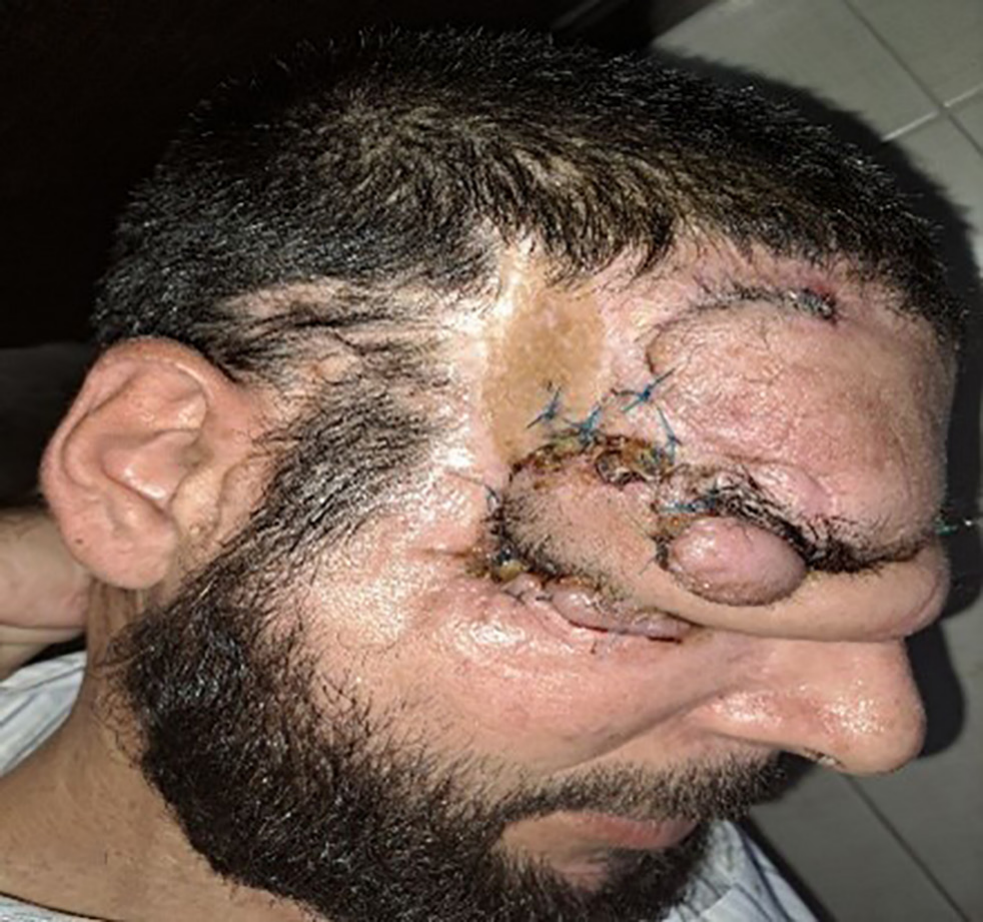
One week postoperatively, a side view showing the flap still attached to its pedicle with the resolution of venous congestion following leech therapy.

**Figure 12.**
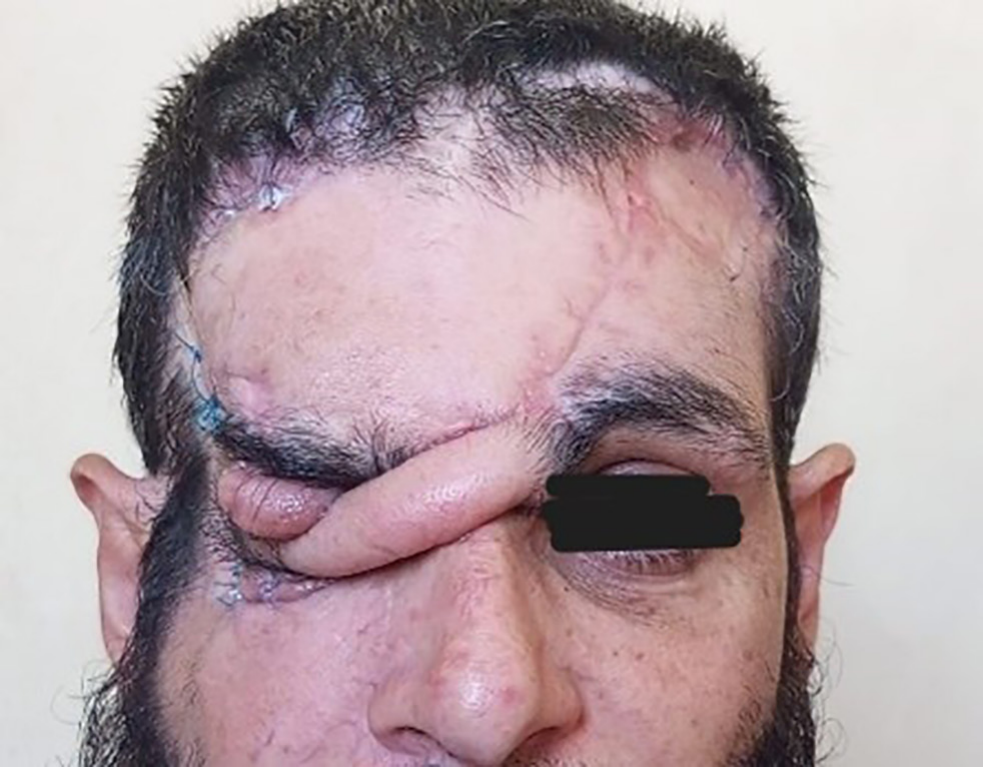
One week postoperatively, a front view showing the flap attached to its pedicle with good viability and resolution of venous congestion after leech therapy.

**Figure 13.**
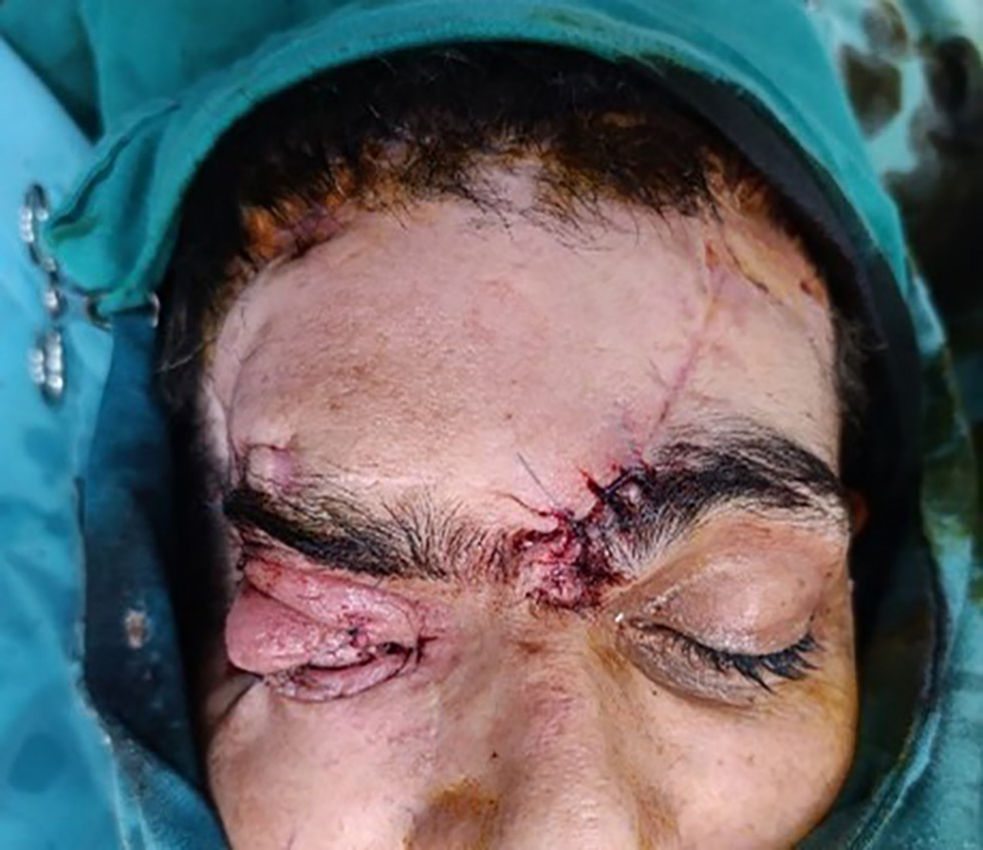
Final stage of the procedure showing the separation of the forehead flap from its pedicle (frontal view).

**Figure 14.**
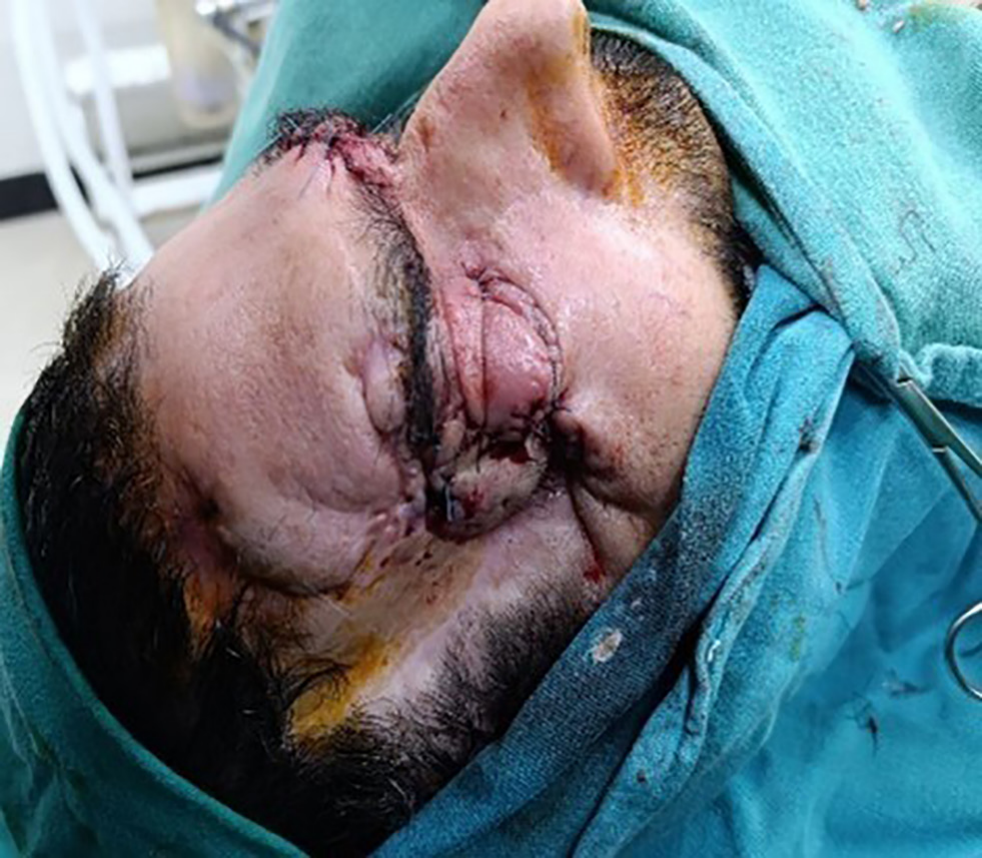
Final stage of the procedure showing the separation of the forehead flap from its pedicle (oblique view).

By the first postoperative week, the flap remained viable and well-perfused, with clear resolution of venous congestion, as shown in the side and front views (Figs. 11 and 12). Three weeks after inset, the flap was successfully separated from its pedicle (Figs. 13 and 14).

At the time of pedicle division, lateral canthus suspension was performed to recreate lateral eyelid support without rigid alloplastic hardware. A small hole was drilled through the implanted iliac crest bone graft, and nonabsorbable nylon sutures, mainly 4-0 and 5-0, were passed through this opening. The sutures were then passed subcutaneously from the lateral ends of the upper and lower eyelids and secured in a loop-like fashion to the bone graft. Tension was adjusted intraoperatively to support the lateral canthus and improve eyelid position without excessive traction. This provided a stable autologous fixation point for the reconstructed lateral canthus and compensated for the absence of conventional rigid fixation hardware.

### 2.4. Postoperative course and imaging

**Figure 15.**
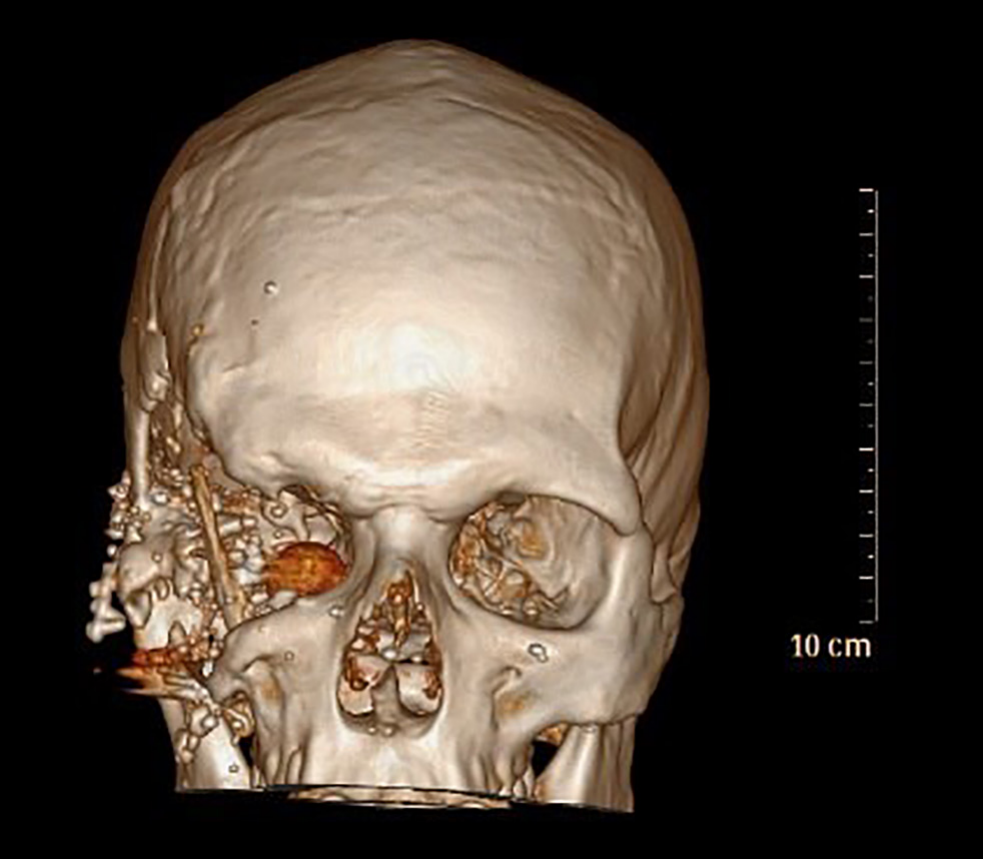
Postoperative 3D CT reconstruction (frontal view) demonstrating the restoration of the right lateral orbital rim with stable positioning of the iliac crest bone graft. 3D = three-dimensional, CT = computed tomography.

**Figure 16.**
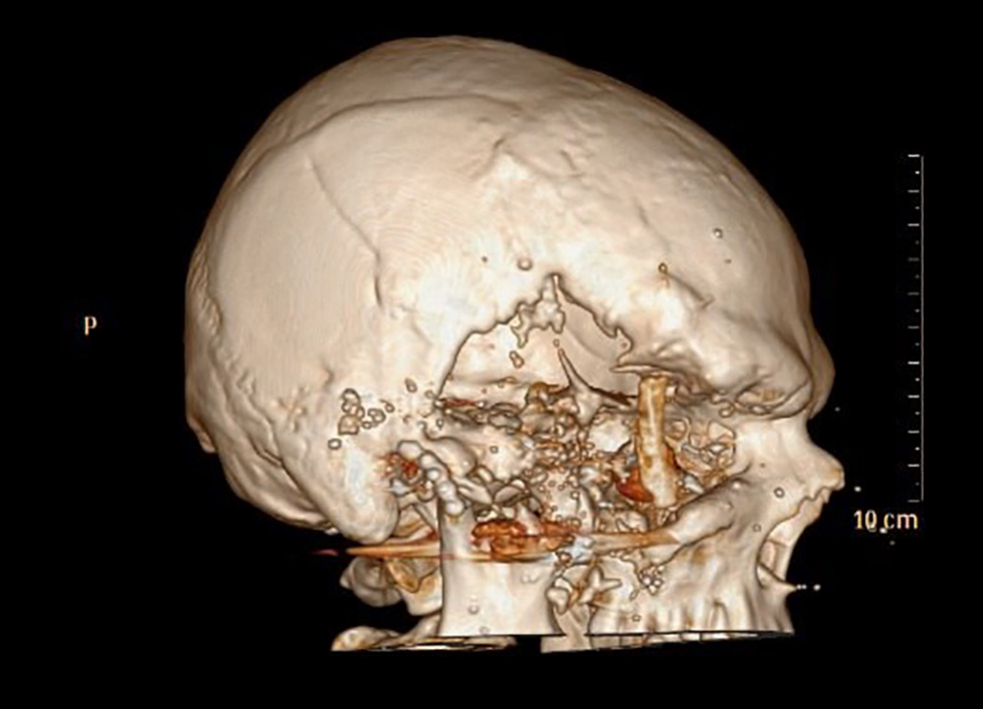
Postoperative 3D CT reconstruction (lateral view) clearly showing the iliac crest graft securely integrated at the reconstructed lateral orbital rim. 3D = three-dimensional, CT = computed tomography.

**Figure 17.**
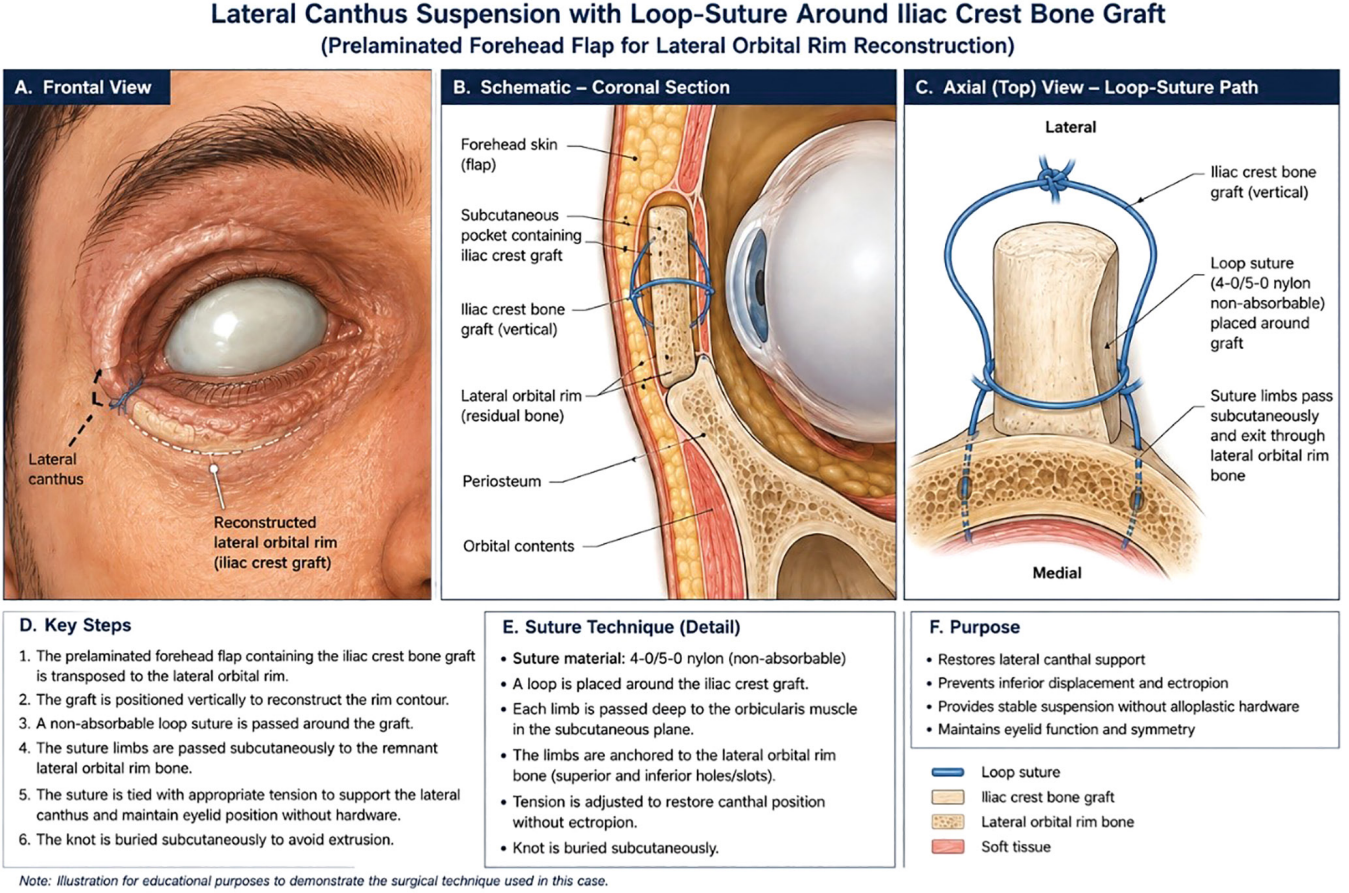
Schematic illustration of the subcutaneous loop-suture lateral canthus suspension technique. The prelaminated contralateral forehead flap containing the iliac crest bone graft is transposed to the lateral orbital rim, and the graft is positioned vertically after rotation to recreate rim support. Nonabsorbable nylon sutures (4-0/5-0) are passed subcutaneously from the lateral upper and lower eyelids and anchored through a small drill hole in the implanted iliac crest graft. The loop-suture is tightened to restore lateral canthal support and improve eyelid position without excessive traction or additional alloplastic hardware.

The postoperative course was uneventful apart from the transient distal venous congestion described above. No partial or complete flap necrosis occurred, and no further vascular compromise was encountered after congestion resolved. Follow-up computed tomography with 3-dimensional reconstruction confirmed appropriate positioning and early stability of the iliac crest graft at the reconstructed lateral orbital rim, demonstrated in frontal and lateral views (Figs. 15 and 16). Because imaging was obtained at 1 month, long-term bone graft resorption could not be assessed at this stage. A schematic illustration of the loop-suture lateral canthus suspension around the implanted iliac crest graft is provided in Figure 17.

The total staged reconstructive course lasted approximately 1 year. Despite the prolonged treatment duration and multiple procedures, the patient tolerated the staged approach well. The reconstruction achieved stable lateral orbital contour, improved facial appearance, and an anatomically adequate socket for future ocular prosthesis placement. The reconstructed socket retained an adequate conjunctival cul-de-sac, which was assessed intraoperatively using a large custom plastic shell conformer simulating an ocular prosthesis; this confirmed sufficient socket depth and retention potential. However, actual ocular prosthesis fitting had not yet been completed because of the patient’s limited financial resources and residence in a distant province, which made access to prosthetic services difficult. Therefore, prosthetic readiness in this case should be interpreted as an anatomical rather than a completed functional outcome. Nevertheless, the patient reported meaningful psychosocial improvement, including improved confidence in social interaction and the ability to appear in public without relying on glasses to conceal the deformity.

## 3. Discussion

Orbital fractures are among the most common midfacial fractures and can result in significant functional impairment,^[3]^ with the third decade of life most frequently affected (29%).^[9]^ Jaquiéry differentiated orbital trauma into 4 classes, from small isolated defects (≈1–2 cm^2^) of the orbital floor or medial wall to defects involving the entire floor and medial wall up to the infraorbital fissure, with the integrity of bony structures at the fissure distinguishing intermediate categories.^[10]^ The timing of surgery remains debated; apart from trapdoor fractures at risk of ischemic contracture, which warrant urgent intervention, several days are generally allowed for orbital and eyelid edema to resolve, enabling a more accurate assessment of extraocular muscle function.^[11]^ Despite the prevalence of post-traumatic orbital injuries, the optimal management pathway remains unsettled, spanning the timing of surgery, operative approach, and implant selection. The choice of implant is influenced by defect complexity and location, surgeon experience and preference, and resource availability.^[12]^ Operative repair may be indicated immediately, for example, in pediatric trapdoor fractures with extraocular muscle entrapment or when a pronounced oculocardiac reflex threatens hemodynamic stability, or deferred according to the persistence or progression of post-traumatic symptoms.^[13]^ Burnstine’s guidelines recommend immediate intervention (<24 hours) when computed tomography demonstrates extraocular muscle or soft tissue entrapment with an oculocardiac reflex, and early repair within 1 to 14 days for persistent symptomatic diplopia or large orbital floor defects.^[14]^ By contrast, our patient underwent late secondary reconstruction 14 years after the initial trauma and failed mesh management; timing was dictated by the eradication of chronic infection and stabilization of local tissues, rather than the immediate or early windows recommended for acute orbital fracture repair.

Unlike acute orbital fractures, this case involved a long-standing composite post-traumatic defect after failed infected alloplastic reconstruction. Therefore, the reconstructive goals were different: infection control, stable soft tissue coverage, bony lateral rim support, lateral canthal stabilization, and creation of an anatomically suitable socket for future ocular prosthesis placement. In this context, a staged autologous strategy was selected to avoid reintroducing rigid alloplastic material into a previously infected field.

Orbital fracture repair entails mobilizing entrapped soft tissues and restoring normal orbital position and volume by bridging the bony defect with stable fixation using an implant.^[7]^ There is likewise no consensus on a single optimal implant for orbital reconstruction.^[15,16]^ Common options include titanium mesh,^[17,18]^ autologous bone grafts,^[19]^ porous polyethylene,^[17]^ titanium–porous polyethylene composites,^[18]^ resorbable materials,^[20–22]^ and preformed computer-assisted orbital implants,^[20]^ each with characteristic advantages and drawbacks, including availability, stability, contourability, radiopacity, donor-site morbidity, rigidity, drainage, and cost.^[15,16]^ Accordingly, implant selection should prioritize features that reduce complications, recognizing that no single material is universally superior.^[15,16]^

In our case, implant choice and technique were dictated by a long-standing composite lateral orbital rim defect, previous infected hardware exposure, limited local tissue quality, and the patient’s need for future prosthetic rehabilitation. The exact novelty of this case lies in bridging the lateral orbital rim defect without additional rigid alloplastic hardware by using a bone-prelaminated pedicled forehead flap. A targeted PubMed search, described in the Introduction, did not identify a prior report of this specific approach for lateral orbital rim reconstruction. The iliac crest graft was placed in direct contact with the underlying galeal/frontalis vascular bed during prelamination, and the narrow graft-matched pocket allowed stability without suture fixation.

An iliac crest graft was selected instead of a calvarial bone graft because of greater institutional and surgeon experience with iliac crest harvesting and limited availability of the specialized instruments required for safe calvarial bone harvest in our setting. The iliac crest also provided a readily contourable graft suitable for recreating lateral orbital rim support while avoiding additional operative complexity in an already traumatized craniofacial region.

A key technical limitation of this approach was the approximately 270° pedicle twist required for flap transfer. This was a true rotational twist rather than a simple 180° transposition arc, and it likely contributed to transient venous congestion by increasing the risk of pedicle kinking, compression, and impaired venous outflow. The congestion resolved after loosening superficial sutures and applying medicinal leech therapy with levofloxacin prophylaxis. When leech therapy is used, antimicrobial prophylaxis targeting Aeromonas species should be administered, using a fluoroquinolone or third-generation cephalosporin according to local protocols and susceptibility patterns.

Bone graft resorption remains an important concern with any non-vascularized autologous graft. In this technique, late resorption would have implications beyond the loss of bony contour because the iliac crest graft also served as the anchoring point for lateral canthus suspension. Significant resorption could weaken loop-suture fixation and may lead to recurrent lateral canthal malposition, ectropion, reduced eyelid support, impaired prosthetic retention, or recurrence of the lateral orbital contour deformity. Therefore, long-term clinical and radiologic follow-up is required to evaluate graft durability and canthal stability.

The staged nature of this reconstruction required multiple procedures over approximately 1 year, which may impose logistical, psychological, and financial burdens. However, in this resource-limited setting, treatment in a public hospital avoided the cost of patient-specific implants or additional alloplastic hardware after prior mesh infection. The reconstruction relied on locally available autologous tissue and standard surgical materials, making it a cost-conscious option when microsurgical reconstruction, patient-specific implants, or repeated alloplastic reconstruction are not feasible.

Although the patient achieved a stable orbital contour, improved facial appearance, and an anatomically suitable socket for future ocular prosthesis placement, actual ocular prosthesis fitting had not yet been completed because of financial and geographic barriers. Therefore, prosthetic readiness should be interpreted as an anatomical outcome rather than a completed functional endpoint. This case remains limited by its single-patient nature, short radiologic follow-up, inability to assess long-term graft resorption, and the absence of completed prosthetic rehabilitation. Longer follow-up is required to determine the durability of the bony reconstruction, canthal suspension, and final prosthetic outcome.

## 4. Conclusion

A staged autologous approach restored stable soft tissue coverage and lateral orbital rim support in a long-standing composite post-traumatic defect within a resource-limited setting. Culture-directed infection control and crane flap preparation created a suitable wound bed, followed by reconstruction using a contralateral forehead flap prelaminated with a subcutaneous iliac crest bone graft. The graft also served as an autologous anchor for subcutaneous loop-suture lateral canthus suspension, avoiding the need for additional rigid alloplastic hardware in a previously infected field. Early postoperative imaging confirmed stable graft positioning, and the reconstructed socket was anatomically suitable for future ocular prosthesis placement. However, actual ocular prosthesis fitting had not yet been completed because of socioeconomic and geographic barriers; therefore, prosthetic readiness should be interpreted as an anatomical rather than a completed functional outcome. This case suggests that bone prelamination of a pedicled forehead flap may represent a cost-conscious reconstructive option when microsurgical reconstruction or patient-specific implants are not feasible, but longer follow-up is required to assess graft durability, canthal stability, long-term contour maintenance, and final prosthetic rehabilitation.

## Author contributions

**Supervision:** Mhd Husam Alhilbawi.

**Validation:** Hussein Hammadeh.

**Writing – original draft:** Ahmed Al-Marrawi, Mohammad Alaa Aldakak.

**Writing – review & editing:** Ahmed Al-Marrawi, Hussein Hammadeh, Mohammad Alaa Aldakak.
